# Evaluation of Prognosticators and Treatment-Related Side Effects in Patients Irradiated Postoperatively for Endometrial Cancer

**DOI:** 10.3390/cancers12123613

**Published:** 2020-12-03

**Authors:** Martin Leu, Jacqueline Possiel, Markus A. Schirmer, Andrea Hille, Stefan Rieken, Leif Hendrik Dröge

**Affiliations:** Department of Radiotherapy and Radiation Oncology, University Medical Center Göttingen, Robert-Koch-Str. 40, 37075 Göttingen, Germany; martin.leu@med.uni-goettingen.de (M.L.); jacqueline.possiel@med.uni-goettingen.de (J.P.); mschirmer@med.uni-goettingen.de (M.A.S.); ahille@med.uni-goettingen.de (A.H.); stefan.rieken@med.uni-goettingen.de (S.R.)

**Keywords:** endometrial cancer, radiotherapy, brachytherapy, teletherapy, prognosticators, grading, nodal stage, age, outcome, side effects

## Abstract

**Simple Summary:**

Several trials sought to improve outcomes in endometrial cancer patients with multimodal strategies. Histopathological, clinical, and molecular tumor characteristics were demonstrated to correlate with outcomes. We tested the hypothesis that specific histopathological and clinical parameters are prognosticators for outcomes in patients irradiated postoperatively at our Gynecological Cancer Center. First, we found a significant influence of grading and nodal stage on outcomes. These findings underline the recommendations of more intense treatment in these patient groups, as reflected in current guidelines. Secondly, age had a significant influence on survival be it due to comorbidities and/or due to too hesitant treatment regimen in elderly. Thus, here, it should be aimed at particular treatment strategies. Lastly, we found low rates of side effects associated with brachytherapy and moderate rates of side effects associated with teletherapy. Overall, our study serves as basis for further improvement of treatment strategies and for conceptualization of clinical trials.

**Abstract:**

Numerous clinical trials sought to improve outcomes in endometrial cancer patients with multimodal treatment strategies. We tested the hypothesis that specific histopathological and clinical parameters are prognosticators for outcomes at our Gynecological Cancer Center. A total of 203 patients (median age, 69.5 years) was included. They were irradiated postoperatively (n = 184: Brachytherapy, n = 19: Teletherapy) between 05/2007 and 03/2020. The median follow-up was 37.2 months. As statistical methods, we used the univariable Cox proportional hazards regression, and log-rank statistics. First, we found a significant influence of grading and nodal stage on outcomes. These findings underline the recommendations of more intense treatment in these patient groups, as already reflected in current guidelines. Secondly, we found that patient age had a significant influence on survival be it due to comorbidities and/or due to too hesitant treatment regimen in the elderly. Thus, it should be aimed at particular strategies in treatment of these patients. Lastly, we found very low rates of treatment-related side effects in patients treated with brachytherapy and moderate rates of side effects in patients treated with teletherapy. Overall, our study serves as basis for further improvement of treatment strategies and for conceptualization of clinical trials.

## 1. Introduction

Numerous larger clinical trials sought to improve both locoregional and distant control in patients with endometrial cancer with elaborated multimodal treatment strategies. Surgical resection, brachytherapy, teletherapy, and systemic treatment are the mainstays of these approaches [1,2,3,4]. In this context, studies demonstrated that several clinical and histopathological characteristics are prognosticators for patient outcomes [5,6]. Additionally, molecular tumor characteristics have recently gained more attention and are currently implemented in ongoing studies to further improve patient selection and to guide individualized treatment of patients with endometrial cancer [7,8]. Overall, clinical decision making in patients remains challenging and has recently been intensively discussed [7,9].

Thus, clinical data will certainly be valuable to improve therapeutic management and to conceptualize future clinical trials. Hence, we tested the hypothesis that specific histopathological and clinical parameters are prognosticators for patient outcomes at our Gynecological Cancer Center.

## 2. Results

### 2.1. Patient Cohort and Treatment

A total of 203 patients were included in this study. The median age was 69.5 years (range, 41.2–89.0 years). They were irradiated postoperatively between 05/2007 and 03/2020. Among them, 184 (90.6%) were treated with brachytherapy and 19 (9.4%) were treated with teletherapy. The median follow up was 37.2 months (range, 1.3–151.3 months, Table 1). During the study period, the chemotherapy regimen (in both cases, q3w) were carboplatin (AUC 5-6 mg*min/mL) combined with paclitaxel (175 mg/m^2^), and cisplatin (50 mg/m^2^) combined with doxorubicin (60 mg/m^2^). A laparoscopic transvaginal hysterectomy and adnexectomy was performed in 51 patients (25.1%). In 152 patients (74.9%), a laparotomy with hysterectomy and adnexectomy was performed. A lymphadenectomy was conducted in 118 patients (58.1%, thereof pelvic and para-aortic dissection in 108 patients [91.5%]).

### 2.2. Outcomes

**Figure 1:**
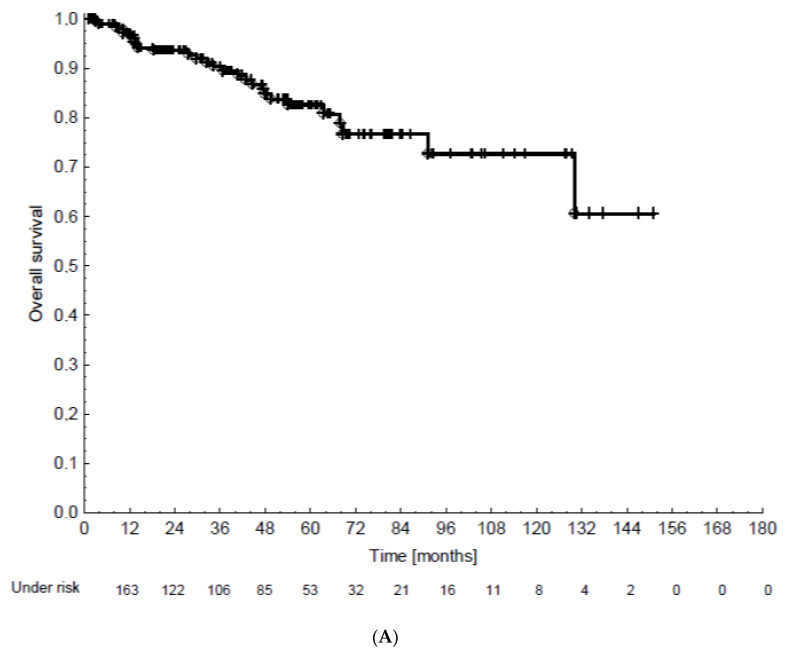

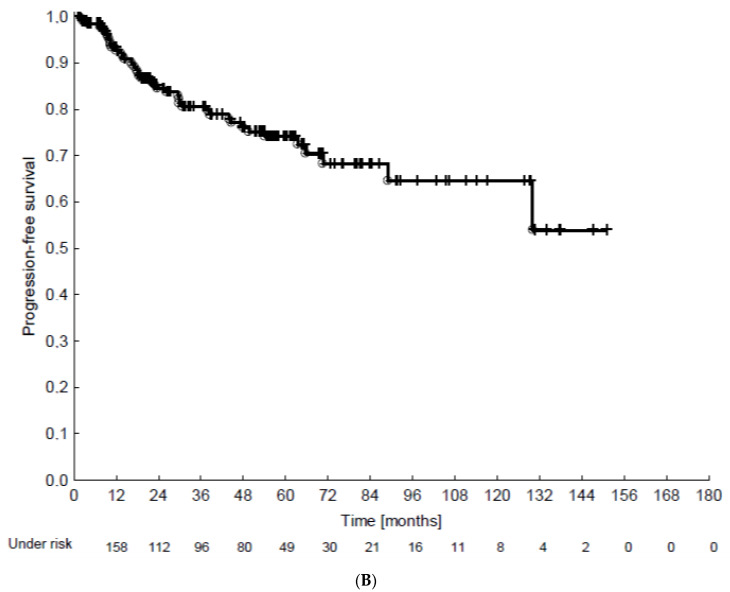

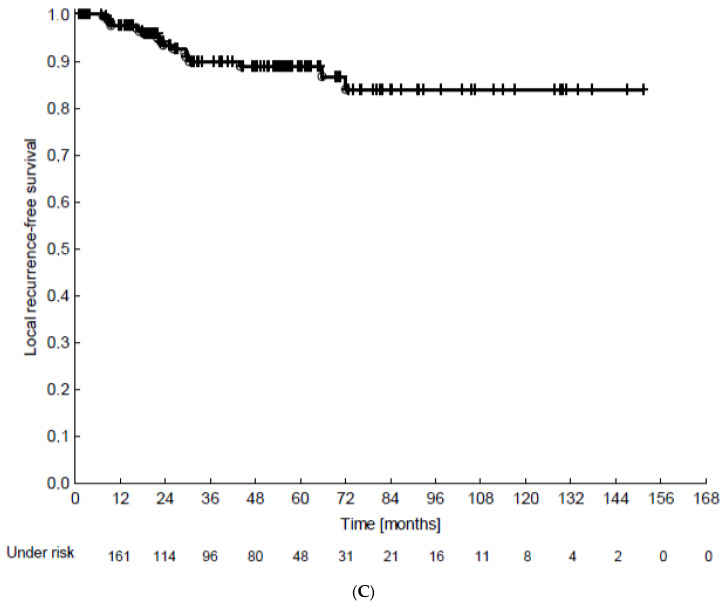
(**A**–**C**): Patient survival in the whole cohort (overall survival, progression-free survival, and locoregional recurrence-free survival).

In the whole patient cohort, the 5-year overall survival (OS), progression-free survival (PFS), and locoregional recurrence-free survival (LRFS) were 82.6%, 74.1%, and 88.9%, respectively (Figure 1A–C). Vaginal recurrences occurred in 7 patients and further pelvic or paraaortic recurrences occurred in 10 patients. During follow-up, distant metastases were found in liver (6 patients), lung (9 patients), cerebrum (4 patients), and bone (4 patients). In total, 28 patients died during follow-up. Among them, 14 patients died from endometrial cancer. In the other patients, the causes of death remained unknown.

### 2.3. Prognosticators

**Figure 2:**
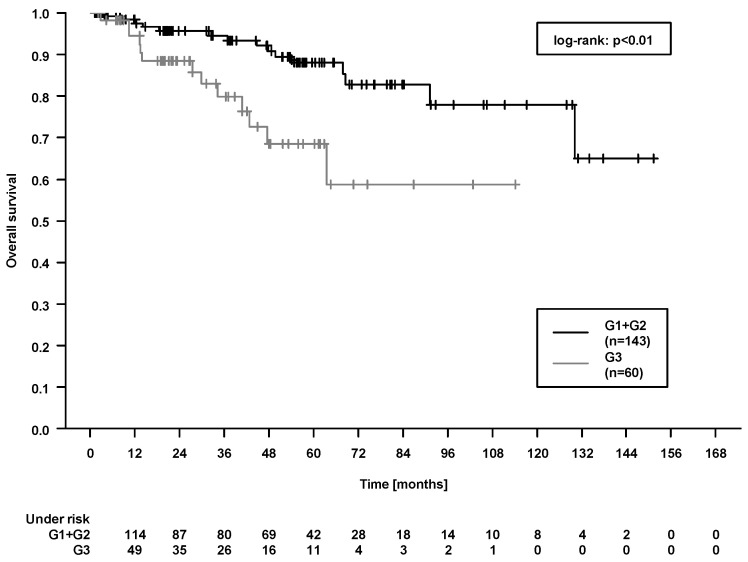
Overall survival in the whole patient cohort, comparison of patients with G1 + G2 tumors vs. patients with G3 tumors.

In the whole patient cohort, in the univariate analysis (at a defined threshold level of *p* < 0.05, Table 2), we identified grading (G3 vs. G1 + G2) associated with all four outcome parameters (LRFS, PFS, OS, and CSS (cancer-specific survival)). Please see Figure 2 for the Kaplan–Meier plot. Similar relations were seen for the second histologic parameter, non-endometrioid vs. endometrioid. Further associations were observed for age (PFS, OS), body mass index (BMI; LRFS, OS), N stage (PFS), lymphatic vessel invasion (PFS, OS), adjuvant chemotherapy (CSS), and Charlson Comorbidity Index (PFS, OS). As we intended to delineate associations of already established parameters and not to identify new ones, the set statistical threshold level of *p* < 0.05 in the univariable Cox regression models was not corrected for multiplicity testing.

### 2.4. Toxicity

In patients treated with brachytherapy, acute organ toxicity ≥ grade 3 occurred in none of the patients. Acute organ toxicity ≥ grade 2 occurred in 2 patients (1.1%). Late toxicity ≥ grade 2 was observed only in one patient (0.5%). In patients treated with teletherapy, acute organ toxicity ≥ grade 3 occurred in one patient (5%), and acute organ toxicity ≥ grade 2 was documented in 7 patients (36.9%). Only one patient (5%) experienced late toxicity ≥ grade 2. Please see Tables S1–S4 for further details.

In the whole cohort, radiotherapy could not be completely applied in 4 patients. The reasons in patients treated with teletherapy were the occurrence of metastasis during treatment (*n* = 1) and patient’s wish (*n* = 1). The reasons in patients treated with brachytherapy were cardiac symptoms in an elderly patient (*n* = 1) and patient’s wish (*n* = 1).

## 3. Discussion

Recently, numerous landmark trials contributed to a refinement of oncological strategies in the treatment of endometrial cancer. Surgical resection is the mainstay of treatment with curative intent. Additive radiotherapy has been demonstrated to be an important component of treatment to achieve a satisfactory local control and to avoid regional recurrences which are often difficult to salvage. Chemotherapy is primarily added to address potential distant metastases [1,2,3,4,10]. The guidelines (German S3 guidelines, [11], ESMO guidelines, [12]) give recommendations for postoperative radiotherapy and, more specifically, for indications of brachytherapy or teletherapy. These recommendations are primarily based on histopathological tumor characteristics [11,12]. Additionally, several studies demonstrated that clinical characteristics are prognosticators for patient outcomes, e.g., patient age and obesity [5,6,13]. Recently, molecular tumor characteristics have gained more and more attention as prognosticators [14]. Current studies have started to implement these molecular characteristics for treatment stratification to improve individualized treatment, e.g., the PORTEC 4a trial [8]. However, for clinicians, decision making remains challenging and the detailed recommendations for treatment-based on tumor stage, histopathological characteristics, molecular characteristics, and clinical factors—is being intensively discussed [7,9]. Thus, clinical data will certainly be valuable to further improve individualized patient treatment. Herein, we tested the hypothesis that specific histopathological and clinical parameters are prognosticators for patient outcomes at our Gynecological Cancer Center.

As strongest prognosticator in our study, grading was associated with LRFS, PFS, OS, and CSS. Previous studies have already described grading as a negative prognostic factor associated with an aggressive biological tumor behavior in patients with endometrial cancer. Especially, high-grade tumors have been reported to be associated with higher tumor stages, the presence of lymph node metastases, the extent of myometrial invasion, and lower patient survival [15]. Thus, the landmark trials during the last decades used tumor grading as an important criterion for patient inclusion [1,2,3,4]. Consequently, clinical guidelines have incorporated tumor grading as a factor to guide individual treatment decisions [11,12]. In detail, previous studies have reported 5-year survival rates of about 70% in patients with G3 tumors [15]. This can be compared to the OS rate at 5 years in patients with high-grade tumors in our study. Remarkably, here, the absolute difference in OS between patients with G1-G2 tumors and patients with G3 tumors amounts to about 20% (Figure 2). Hence, our results highlight the relevance of tumor grading for therapeutic decisions and demonstrate the need to make efforts to improve patient outcomes in this group with multimodal treatment strategies.

Additionally, we found nodal status (N+ [FIGO stages IIIC1-2, [16]] vs. N0 status) to be of prognostic relevance for the patients in case of PFS. Previous studies had demonstrated that nodal metastases are a very important risk factor in endometrial cancer patients [7]. However, clinical studies have recently raised the question, whether and to what extent patients should undergo lymphadenectomies [7,17]. This decision is often made depending on individual tumor-related risk factors. At the same time, surgical complication rates are influenced by the present comorbidities [11,18,19]. Here, it should be mentioned, that, in our study, 67.0% of the patients were overweight (body mass index ≥ 25) and 81.3% of the patients had relevant comorbidities (Charlson Comorbidity Index ≥ 4). Generally, overweight patients seem to have a fairly good prognosis. In our study, we found increased BMI beneficially related to LFRS (for a BMI ≥ 30) and OS (for a BMI ≥ 25). Modesitt et al. [13] have reported a lower BMI in more aggressive histologies and advanced stages conclusive with tumor cachexia. Accordingly, in our data, a relation of a low BMI with non-endometrioid and G3 tumors was also seen. These aspects should be considered when balancing the surgery-related risks and oncological benefits in overweight patients, too. Altogether, in our study, 118/203 patients (58.1%) received a lymphadenectomy. This can be compared to the recent PORTEC 3 trial collective with—depending on the study arm—lymphadenectomy in about 55–60% of all patients [3]. As a major result, the PORTEC 3 trial demonstrated that especially patients with FIGO stages III (here, more than half of the patients with N+ status [FIGO stage IIIC]) had an absolute improvement of 10% in 5-year OS with chemoradiotherapy versus radiotherapy alone [3,20]. With a hazard ratio of 2.68 in case of PFS when comparing N+ vs. N0 status, our study results underline the worse outcomes in these patients. However, it should be noted that there were only 14 patients with N+ status in our cohort. Overall, on the one hand, the indication of lymphadenectomy should be discussed carefully in the light of the aforementioned aspects (overweight, comorbidities). On the other hand, the knowledge of the exact tumor stage is important to form a basis for therapeutic decisions and to intensify local and systemic treatment, if necessary. However, multidisciplinary treatment decisions remain challenging in these patients with advanced endometrial cancer.

In addition, we found patient age as a significant prognosticator in terms of PFS and OS. Previous studies had already reported an association of patient age and survival endpoints in endometrial cancer [6,21]. However, patient age does not alter therapeutic management in the current guidelines, yet [6,11,12]. As previous authors reported, the poor prognosis in elderly patients can be explained by the occurrence of more aggressive tumors [22,23]. However, our data did not support any relation between histopathologic parameters and patient age, i.e., the distribution of high-grade malignancies was not affected by patient age. The observed association of age with OS most likely reflects the simple inverse relationship between age and remaining life expectancy. This aspect is partly reflected in our study, since we found the Charlson Comorbidity Index to be associated with PFS and OS. Consistently, the presence of relevant comorbidities might worsen the prognosis [6]. In detail, in elderly patients with increased comorbidities, an excessive risk associated with vaginal bleeding caused by the primary tumor or increased intraoperative and perioperative complications might cause the higher mortality rates. Moreover, especially since multimodal therapies are often associated with higher rates of side effects, elderly patients might be less likely to successfully complete all components of treatment [23]. On the other hand, as aggressive tumors in the elderly are as frequent as in younger patients, studies indicate that treatment intensity might be too reluctant in the advanced age group [6,23]. Recent studies reported that especially older patients benefit from more aggressive multimodal strategies. In the PORTEC-3 trial, in multivariate analysis, women ≥ 70 years derived the greatest benefit from chemoradiotherapy in terms of failure-free survival [20]. Overall, in the light of an often aggressive tumor behavior and the other aforementioned factors, the clinical decision making is challenging in elderly patients. Besides, these patients are rarely included in clinical trials. In the PORTEC-3 trial, only about 20% of the patients were ≥70 years old. Hopefully, future studies will take these aspects into account [24].

As mentioned before, the patient cohort in our study included a relevant proportion of elderly patients and patients with relevant comorbidities. These conditions predispose for treatment morbidity [25]. In addition, a previous publication from our institution had indicated that, in patients irradiated for endometrial cancer, the occurrence of acute toxicities is correlated with the occurrence of late toxicities [26]. Both aspects underline that a thorough discussion of the rates of treatment-related side effects should be made. With regard to vaginal brachytherapy, in recent literature, the rates of toxicities are reported to be very low ([27], in 0–5.2% of all patients). It has been reported that especially lower doses per fraction are associated with the lowest rates of side effects [27]. In our study, the rates of ≤ 2% of patients with ≥ grade 2 toxicities (both acute and late) were favorable and comparable to the previously reported rates [27]. Accordingly, in our study, a fairly low dose per fraction (2.5–5.0 Gy) might have contributed to the very low toxicity rates [27]. Finally, as previously reported by our study group, a further refinement of vaginal brachytherapy techniques (here, the consideration of the bladder filling and the exact applicator position) might further improve toxicity profiles [28]. With regard to teletherapy, modern series reported acute toxicity rates of ≥ grade 2 in about 25% of the patients and late toxicity rates of ≥ grade 2 in about 5% the patients [29]. In our study, acute toxicity ≥ grade 2 occurred in 36.9% of all patients and late toxicity ≥ grade 2 was documented in 5 % of the patients. Thus, we observed slightly higher rates of acute toxicities than Chen et al. [29]. This might be explained by the fact that we used modern radiotherapy techniques (IMRT/VMAT) in only 63.1% of all patients, while Chen et al. used these techniques in all patients [29]. The rate of late toxicities was comparable with ≥ grade 2 in 5% of the patients in our study. Here, the results should be interpreted carefully, since in our study, only 14/19 patients attended follow-ups and were documented in late toxicity assessments. In conclusion, in teletherapy of endometrial cancer, to avoid excessive morbidity, and, consequently, to leave space for optimization of multimodal concepts, modern radiotherapy techniques like IMRT or VMAT should be standardly applied. In modern studies, these modern radiotherapy techniques have already been implemented into the study protocols [8].

Finally, our study’s limitations should be briefly discussed. First, we present a retrospective single-institution study over an inclusion period of 13 years. Thus, treatment was conducted by different physicians, and, especially since several important clinical trials on endometrial cancer had been reported during this period [2,3,4], treatment patterns have changed over time. Consequently, it can be assumed that patients were not treated completely homogeneously. Secondly, as the proportion of patients treated with teletherapy was fairly small, an extended cohort with more patients treated with teletherapy could give further insights. Thirdly, in 41.9% of the patients, lymphadenectomies were not performed. Thus, a relevant proportion of patients might have been understaged. This might have had an impact on postoperative treatment strategies. Additionally, this might represent a relevant bias for our study’s results. Overall, we analyzed a relatively large cohort of patients with endometrial cancer treated using mostly modern irradiation techniques at a tertiary cancer center. This is why our study contributes to current understanding of the clinical courses of endometrial cancer patients.

## 4. Materials and Methods

### 4.1. Patients

We retrospectively reviewed the medical records of patients who were irradiated postoperatively at our institution for endometrial cancer in curative intent. Patients with distant metastases were excluded from the analysis. The staging examinations and the further procedures during multimodal oncological treatment were performed according to the current guidelines [11] at our Gynecological Cancer Center. The staging examinations included an abdominal ultrasound and a chest radiograph, or a computed tomography scan of the chest and abdomen. A rectoscopy or cystoscopy was performed in patients with clinical or radiological suspicion of invasion of these organs. This study was approved and the requirement to obtain additional informed consent was waived by the ethic committee of the University Medical Center of Göttingen (application number 27/9/20). The study has been conducted in accordance with the Declaration of Helsinki principles.

### 4.2. Brachytherapy and Teletherapy

The high dose rate brachytherapy was applied up to a total dose of 20 Gy in 5 Gy fractions or to a total dose of 15 Gy in 2.5 Gy fractions (see Table 1 for patient distribution). The treatment was performed on an outpatient basis. An appropriate applicator (Elekta, Nucletron, Sweden) was placed in the vagina. Afterwards, pelvic computed tomography scans were acquired (Brilliance, Big Bore Oncology, Philips, Netherlands). For irradiation, a Nucletron afterloader was used (microSelectron HDR, Elekta, Sweden, Iridium-192 source). The dose was prescribed to a depth of 5 mm from the surface of the vaginal applicator. The decision about the length of the vagina which was supposed to be irradiated was left at the discretion of the treating physician. Please see Bergau et al. [28] for further details.

The teletherapy was applied with linear accelerators and daily image guidance (Varian Medical Systems Clinac 2300 CD-3376 and 2300 CD-3686, photons with 6 MeV or 20 MeV). Patients were instructed to present with a comfortably filled bladder and received a planning CT scan with a slice thickness of 5 mm. The guideline by Small et al. served as reference for contouring of clinical target volumes [30]. Accordingly, the common iliac, external iliac, internal iliac and presacral lymph nodes were standardly included [30]. Additionally, according to the guidelines, the upper vagina and the parametrial and paravaginal tissue were standardly included [30]. The paraaortic lymph nodes were included in cases when tumor infiltration was documented in this region. For the planning target volume, a 10 mm margin was added to the clinical target volume. 3D conformal radiotherapy (4 pelvic fields), intensity-modulated radiotherapy (IMRT) or volumetric modulated arc therapy were used (see Table 1 for patient distribution). The planning system Eclipse (Varian Medical Systems) was used for dose calculation.

### 4.3. Follow-Up and Toxicity Scoring

During radiotherapy, weekly assessments were conducted by the treating physicians in our clinic, including the taking of blood samples. After radiotherapy, the follow-up examinations in our clinic were regularly conducted every 18 months for at least 5 years. Additionally, patients had regular follow-up examinations by the treating gynecologist. We used the Common Terminology Criteria for Adverse Events (current version 5.0) [31] and the Late Effects on Normal Tissues/Subjective, Objective, Management, and Analysis score [32] to assess the acute and late toxicities, respectively.

### 4.4. Statistics

Patient baseline, tumor and treatment data were analyzed with respect to LRFS, PFS, OS, and CSS by means of univariable Cox proportional hazards regression. Survival times were calculated from the day of pathologically determined malignancy diagnosis until the end of follow-up. LRFS was considered as the time from diagnosis to local recurrence or to recurrence in pelvic or paraaortic lymph nodes. PFS was considered as the combined endpoint of time to locoregional or distant recurrence and death from any cause. CSS was the time to death due to endometrial cancer. Visual illustration of Figure 1 and Figure 2 was performed by Kaplan–Meier plots along with log-rank statistics. Data were analyzed using the software SPSS Statistics (v 26, IBM) and R 4.0.2 with “KMWin” (Kaplan–Meier for Windows) plugin [33].

## 5. Conclusions

Numerous larger clinical trials sought to improve outcomes in patients with endometrial cancer with elaborated multimodal treatment strategies. Histopathological, clinical, and—recently—molecular tumor characteristics have been demonstrated to correlate with outcomes [5,6]. However, clinical decision making remains challenging [7,9]. Herein, we tested the hypothesis that specific histopathological and clinical parameters are prognosticators for patient outcomes at our Gynecological Cancer Center. Firstly, we found an influence on clinical outcome parameters for grading (G3 vs. G1-2, LRFS, PFS, OS, and CSS). Nodal stage (N+ vs. N0) was a prognosticator in case of PFS. These findings underline the recommendations of more intense treatment strategies in these patient groups, as already reflected in current guidelines. Secondly, we found that patient age had a significant influence on survival be it due to comorbidities and/or due to too hesitant treatment regimen in the elderly. Thus, it should be aimed at particular strategies in treatment of these patients. Lastly, we found very low rates of treatment-related side effects in patients treated with brachytherapy and moderate rates of side effects in patients treated with teletherapy. Overall, our retrospective study serves as a basis for further improvement in treatment strategies and for further conceptualization of clinical trials.

## Figures and Tables

**Table 1:** Baseline patient and disease characteristics.

Patient and Disease Characteristics, N (%)	203
Age, median (min–max)	69.5 years (41.2–89.0)
Behavioral factors	
Smoking w/o regular alcohol	25 (12.3)
Alcohol abuse w/o smoking	3 (1.5)
Smoking and alcohol abuse	1 (0.5)
Neither smoking nor regular alcohol	174 (85.7)
T category	
pT1a	30 (14.8)
pT1b	104 (51.2)
pT1c	14 (6.9)
pT2	30 (14.9)
pT3	18 (8.9s)
pT4	2 (0.9)
ypT1a	1 (0.5)
ypT1b	1 (0.5)
ypT2	1 (0.5)
ypT3	2 (0.9)
Nodal status	
pN0	104 (88.1)
pN+	14 (11.9)
FIGO classification	
I	148 (72.9)
II	24 (11.8)
III	29 (14.3)
IV	2 (1.0)
Former history of endometrial cancer	
Yes	3 (1.5)
Chemotherapy	
Adjuvant	
Yes	34 (16.7)
Carboplatin/Paclitaxel	30 (88.2)
Cisplatin/Doxorubicin	4 (11.8)
Incomplete	7 (20.6)
Perioperative	
Yes	5 (2.5)
Carboplatin/Paclitaxel	5 (100)
Incomplete	1 (20)
Radiotherapy	
Brachytherapy	184 (90.6)
Dose, median (min–max)	20 Gy (10–20)
Incomplete	2 (1.1)
Total dose 20 Gy, single fraction 5 Gy	161 (87.5)
Total dose 15 Gy, single fraction 2.5 Gy	23 (12.5)
Irradiation of >the upper third of the vagina	159 (86.4)
Irradiation of ≤the upper third of the vagina	25 (13.6)
Teletherapy	19 (9.4)
Radiotherapy technique	
3D conformal radiotherapy	7 (36.9)
IMRT	2 (10.5)
VMAT	10 (52.6)
Dose, median (min–max)	50.4 Gy (39.6–60)
Incomplete	2 (10.5)
Surgery	
Modality	
Laparoscopic transvaginal hysterectomy and adnexectomy	51 (25.1)
Laparotomy, hysterectomy and adnexectomy	152 (74.9)
Lymphadenectomy	
Yes	118 (58.1)
Pelvic	10 (8.5)
Pelvic + para-aortic	108 (91.5)
No	85 (41.9)
Histology	
Endometrioid adenocarcinoma	176 (86.7)
Serous carcinoma	13 (6.5)
Small cell carcinoma	1 (0.5)
Clear cell carcinoma	7 (3.4)
Endometrioid adenocarcinoma with squamous differentiation	1 (0.5)
Squamous cell carcinoma	1 (0.5)
Tubulo-papillary adenocarcinoma	1 (0.5)
Undifferentiated carcinoma	1 (0.5)
Dedifferentiated carcinoma	2 (0.9)
Grading	
G1	17 (8.4)
G2	126 (62.1)
G3	60 (29.5)
Lymphatic vessel invasion	
Yes	16 (7.9)
No	187 (92.1)
Venous invasion	
Yes	8 (3.9)
No	195 (96.1)
Body mass index	
<25	40 (19.7)
≥25	136 (67.0)
Not available	27 (13.3)
Charlson Comorbidity Index	
1–3	38 (18.7)
4–6	151 (74.4)
7–10	14 (6.9)

**Table 2:** Prognosticators in the whole patient cohort, univariable Cox regression analysis. Numbers of patients are indicated in brackets.

Variable	LRFS		PFS		OS		CSS	
	Hazard Ratio (95% CI)	*p* Value	Hazard Ratio (95% CI)	*p* Value	Hazard Ratio (95% CI)	*P* Value	Hazard Ratio (95% CI)	*p* Value
Age (203), per year	1.03 (0.98–1.09)	0.23	1.07 (1.03–1.11)	<0.01	1.08 (1.03–1.13)	<0.01	1.01 (0.96–1.07)	0.63
BMI								
≥25 (136) vs. <25 (40)	0.52 (0.19–1.40)	0.19	0.68 (0.34–1.37)	0.28	0.41 (0.18–0.91)	0.03	0.36 (0.12–1.04)	0.06
≥30 (76) vs. <30 (100)	0.26 (0.07–0.89)	0.03	0.72 (0.37–1.38)	0.32	0.72 (0.32–1.63)	0.43	0.52 (0.16–1.65)	0.26
FIGO classification								
FIGO ≥ III (31) vs. ≤II (172)	1.65 (0.54–5.05)	0.38	1.75 (0.86–3.57)	0.12	1.24 (0.47–3.27)	0.67	1.49 (0.42–5.35)	0.54
Histology								
Non endometrioid (27) vs. endometrioid (176)	2.51 (0.88–7.12)	0.08	2.02 (0.99–4.12)	0.05	2.27 (0.96–5.38)	0.06	3.52 (1.18–10.53)	0.02
N stage								
N+ (14) vs. N0 (104)	2.98 (0.79–11.25)	0.11	2.68 (1.06–6.62)	0.04	1.91 (0.53–6.86)	0.31	2.61 (0.69–9.85)	0.16
Grading								
G3 (60) vs. G2 (126)	2.44 (0.93–6.43)	0.07	2.65 (1.42–4.93)	<0.01	2.70 (1.25–5.82)	0.01	5.21 (1.71–15.92)	<0.01
G3 (60) vs. G1 + G2 (143)	2.73 (1.04–7.20)	0.04	2.97 (1.59–5.53)	<0.01	3.01 (1.40–6.49)	<0.01	5.79 (1.90–17.67)	<0.01
Lymphatic vessel invasion								
L1 (16) vs. L0 (187)	2.19 (0.50–9.62)	0.30	2.75 (1.15–6.58)	0.02	3.01 (1.03–8.81)	0.04	2.98 (0.65–13.58)	0.16
Adjuvant chemotherapy								
Yes (39) vs. No (164)	1.61 (0.57–4.58)	0.37	1.64 (0.84–3.23)	0.23	1.72 (0.75–3.95)	0.20	3.10 (1.07–8.99)	0.04
Charlson Comorbidity Index								
>5 (50) vs. ≤5 (153)	1.04 (0.34–3.20)	0.94	2.35 (1.27–4.36)	<0.01	3.13 (1.49–6.60)	<0.01	0.98 (0.27–3.53)	0.99
Brachytherapy								
> upper third of the vaginaYes (159) vs. no (25)	0.99 (0.13–7.67)	0.99	0.99 (0.23–4.21)	0.99	1.04 (0.14–7.91)	0.97	0.62 (0.08–4.98)	0.65
Lymphadenectomy								
Yes (118) vs. no (85)	1.04 (0.38–2.82)	0.94	0.72 (0.39–1.32)	0.29	0.58 (0.28–1.22)	0.15	2.14 (0.60–7.69)	0.24
Radiotherapy technique								
Brachytherapy (184) vs. Teletherapy (19)	23.04 (0.01–>100) *	0.42	0.40 (0.19–0.87))	0.02	0.34 (0.14–0.84)	0.02	0.51 (0.11–2.31)	0.39

LRFS = locoregional recurrence-free survival, PFS = progression-free survival, OS = overall survival, CSS = cancer-specific survival. * Hazard ratio not reasonably applicable in view of extensive confidence interval as there were 17 out of 184 and 0 out of 19 LRFS events in the brachytherapy and teletherapy group, respectively. Note that brachytherapy and teletherapy were mutually exclusively applied in our study cohort.

## Supplementary Materials

The following are available online at https://www.mdpi.com/2072-6694/12/12/3613/s1, Table S1.: Acute toxicity in patients treated with brachytherapy. Table S2. Late toxicity in patients treated with brachytherapy. Table S3. Acute toxicity in patients treated with teletherapy. Table S4. Late toxicity in patients treated with teletherapy.
