# Supplementary material for: Evaluation of Prognosticators and Treatment-Related Side Effects in Patients Irradiated Postoperatively for Endometrial Cancer
Source: Cancers (Basel). 2020 Dec 3;12(12):3613. doi: 10.3390/cancers12123613 (PMC7761733; doi:10.3390/cancers12123613)
Supplement: Supplementary file 1 [file cancers-12-03613-s001.pdf]

cancers

Supplementary Materials

Evaluation of Prognosticators and Treatment-Related Side Effects in Patients Irradiated Postoperatively for Endometrial Cancer

Martin Leu, Jacqueline Possiel, Markus A. Schirmer, Andrea Hille, Stefan Rieken and Leif Hendrik Dröge *

Table S1. Acute toxicity in patients treated with brachytherapy.

	0°	I°	II°	III°	IV°
Skin reaction	182	2	0	0	0
Cystitis	179	4	1	0	0
Proctitis	181	3	0	0	0
Enteritis	183	0	1	0	0
Vaginal dryness	178	5	1	0	0

Table S2. Late toxicity in patients treated with brachytherapy.

	0°	I°	II°	III°	IV°
Skin	149	0	0	0	0
Cystitis	146	3	0	0	0
Proctitis	148	1	0	0	0
Vaginal adhesion	147	1	1	0	0
Fistula	149	0	0	0	0
Enteritis	159	0	0	0	0
Lymphedema	148	1	0	0	0

Table S3. Acute toxicity in patients treated with teletherapy.

	0°	I°	II°	III°	IV°
Skin reaction	7	10	2	0	0
Cystitis	8	9	1	1	0
Proctitis	7	9	3	0	0
Enteritis	13	4	2	0	0
Vaginal dryness	17	1	1	0	0

Table S4. Late toxicity in patients treated with teletherapy.

	0°	I°	II°	III°	IV°
Skin	14	0	0	0	0
Cystitis	13	1	0	0	0
Proctitis	13	1	0	0	0
Vaginal adhesion	14	0	0	0	0
Fistula	14	0	0	0	0
Enteritis	14	0	0	0	0
Lymphedema	13	0	1	0	0
